# An unusual presentation of penetrating bladder injury with vesicocutaneous fistula: a case report

**DOI:** 10.1186/s12894-023-01254-1

**Published:** 2023-05-03

**Authors:** Jay Lodhia, Alex Mremi, Bahati Robert, Adnan Sadiq, Frank bright, Orgeness Jasper Mbwambo, Bartholomeo Nicholaus Ngowi

**Affiliations:** 1 Department of General Surgery, Kilimanjaro Christian Medical Centre, PO Box 3010, Moshi, Tanzania; 2 Faculty of Medicine, Kilimanjaro Christian Medical University College, PO Box 2240, Moshi, Tanzania; 3 Department of Pathology, Kilimanjaro Christian Medical Centre, PO Box 3010, Moshi, Tanzania; 4 Department of Radiology, Kilimanjaro Christian Medical Centre, PO Box 3010, Moshi, Tanzania; 5 Department of Urology, Kilimanjaro Christian Medical Centre, PO Box 3010, Moshi, Tanzania

**Keywords:** Foreign body, Thigh abscess, Visicocutaneous fistula

## Abstract

**Background:**

Blunt trauma to the urinary bladder is common with penetrating injury being a rare occasion. Most common entry pint for penetrating injuries includes buttock, abdomen and perineum with thigh being rare. There are a number of complications that may develop as a result of penetrating injury with vesicocutanous fistula being a rare occurrence that usually presents with typical sign and symptoms.

**Case presentation:**

We present a rare case of penetrating bladder injury through medial upper thigh as an entry point that had complicated into vesicocutaneous fistula with atypical presentation of long-standing pus discharge that had been managed by incision and drainage several times with no success. MRI demonstrated a presence of fistula tract and a foreign body (piece of wood) in-situ confirmed the diagnosis.

**Conclusion:**

Fistulas are a rare complication of bladder injuries and can cause negative impact on the quality of life of patients. Delayed urinary tract fistulations and secondary thigh abscesses are uncommon therefore a high index of suspicion is needed for early diagnosis. This case emphasizes the importance of radiological tests in aiding the diagnosis and ultimately proper management.

## Introduction

Bladder injuries can be classified as blunt or penetrating. Generally the bladder is protected but as it fills up, it rises up into the lower abdomen making it more susceptible to injury [1]. Blunt bladder injuries are rather common, however, penetrating injuries to the bladder accounts for 14–49%. The most common entry points are the anterior abdomen, rectum and buttocks, hence injuries via the inner thighs are rare therefore clinicians should have a high index of suspicion for bladder injury regardless of signs and symptoms in such patients, and should be fully evaluated [1, 2].

Thigh abscess can be a sign of intra-abdominal pathologies like diverticulitis or colorectal cancer, however less frequently this can arise from the urinary bladder [3]. A fistula is an abnormal communication between two epithelial lined surfaces [4]. Vesicocutaneious fistula (VCF) can be acquired or congenital characterized by an abnormal tract formed between the urinary bladder and cutaneous surface of the body [5]. Diagnosis can sometimes be challenging due to the obscure clinical history and presentation hence higher modality contrast studies like CT-scan or MRI should be done, as will also facilitate in the plan of management. Herein we present an uncommon cause of vesicocutaneous fistula (VCF) in a school girl who presented with a long-term sinus on her right inner thigh.

## Case presentation

A 16-year-old female patient presented to our centre with a non-healing wound on her right inner thigh for a year and half. She reported that she sustained injury to that area after a fall off a double-decked wooden bed at her boarding school and sustaining injury on her right inner thigh on hitting its corner with minimal bleeding and the wound healed initially within some days. She did not sustain any other injuries and was attending her school normally. She then subsequently had on and off pus discharge with occasional swelling on her right inner thigh that had been incised and drained on two occasions at a nearby health centre with little relief and recurrence. Throughout that period she reported normal urinary habit, normal bowel habit, occasional low-grade fevers, and good appetite with no history of body weight loss. Neither history of diabetes mellitus nor hypertension were reported and no known history of malignancy in the family.

**Fig. 1:**
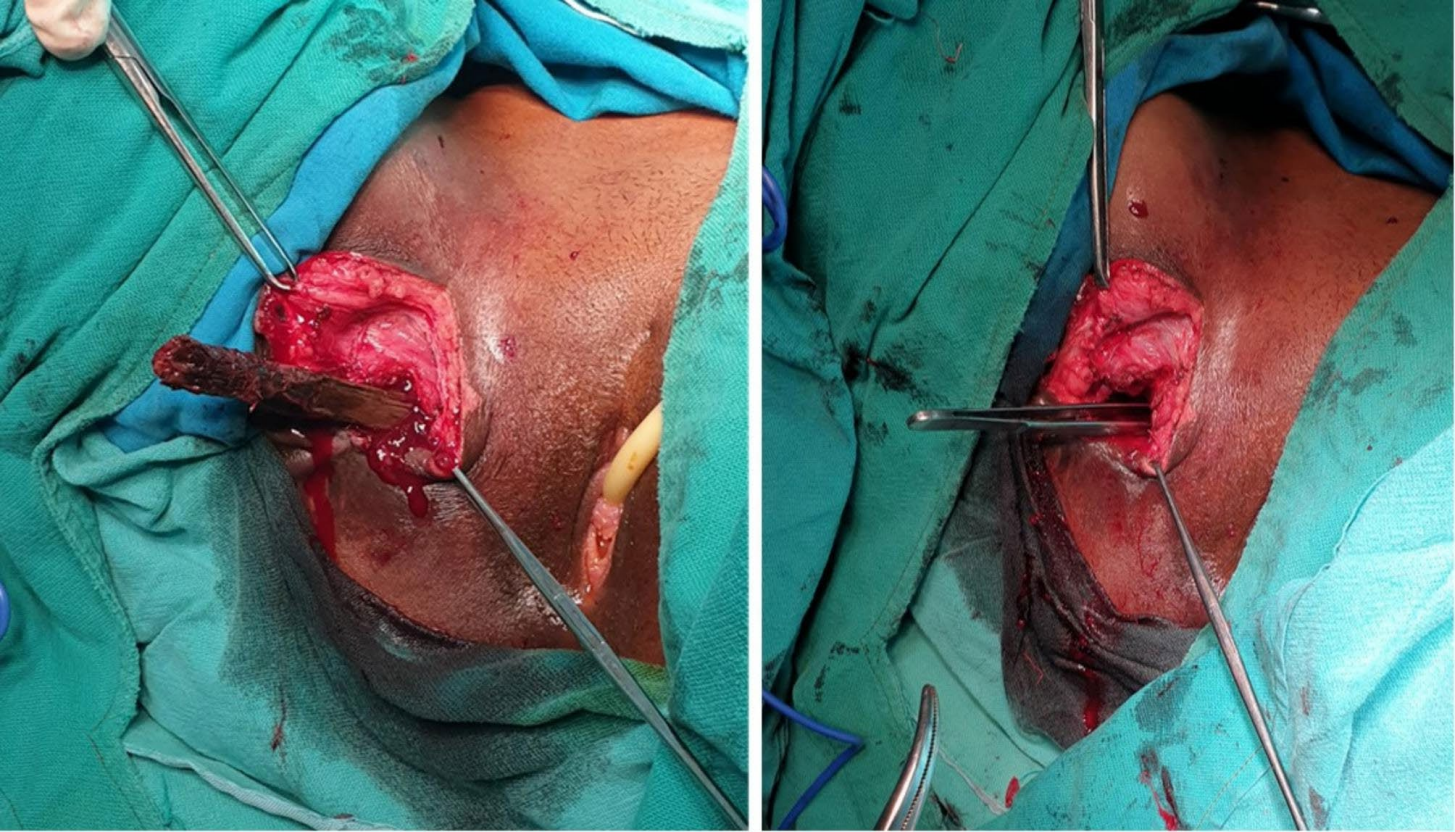
MR images of the pelvis and proximal right thigh – T2 and T2 fat sat images show a fistulous tract measuring 10 cm in length with a foreign body within the tract originating from right lateral wall of the urinary bladder (images A and B) passing through obturator internis, obsturator externus, adductor magnus and gracilis muscles with tract exiting through the medial aspect of right thigh (images D and E). Images C and F shows the foreign body in the fistulous tract (blue arrow)

On examination she was fully conscious, afebrile, not pale, not jaundiced, not dehydrated, nutritionally sound with stable vitals. Local examination revealed a right upper medial thigh pus-discharging sinus, 1х1 cm with exophytic granulomatous margins with scar tissue around. Chronic sinus was then suspected and a pelvic X-ray was ordered that revealed normal osseous outlines. An MRI was requested and reported a fistulous tract measuring about 10 cm in length with a foreign body originating from right lateral wall of the urinary bladder passing through pectineus, adductor longus, adductor brevis, adductor magnus and gracilis muscles exiting through medial aspect of right thigh; in keeping with vesico-cutaneous fistula with a foreign body in-situ (Fig. 1).

**Fig. 2:**
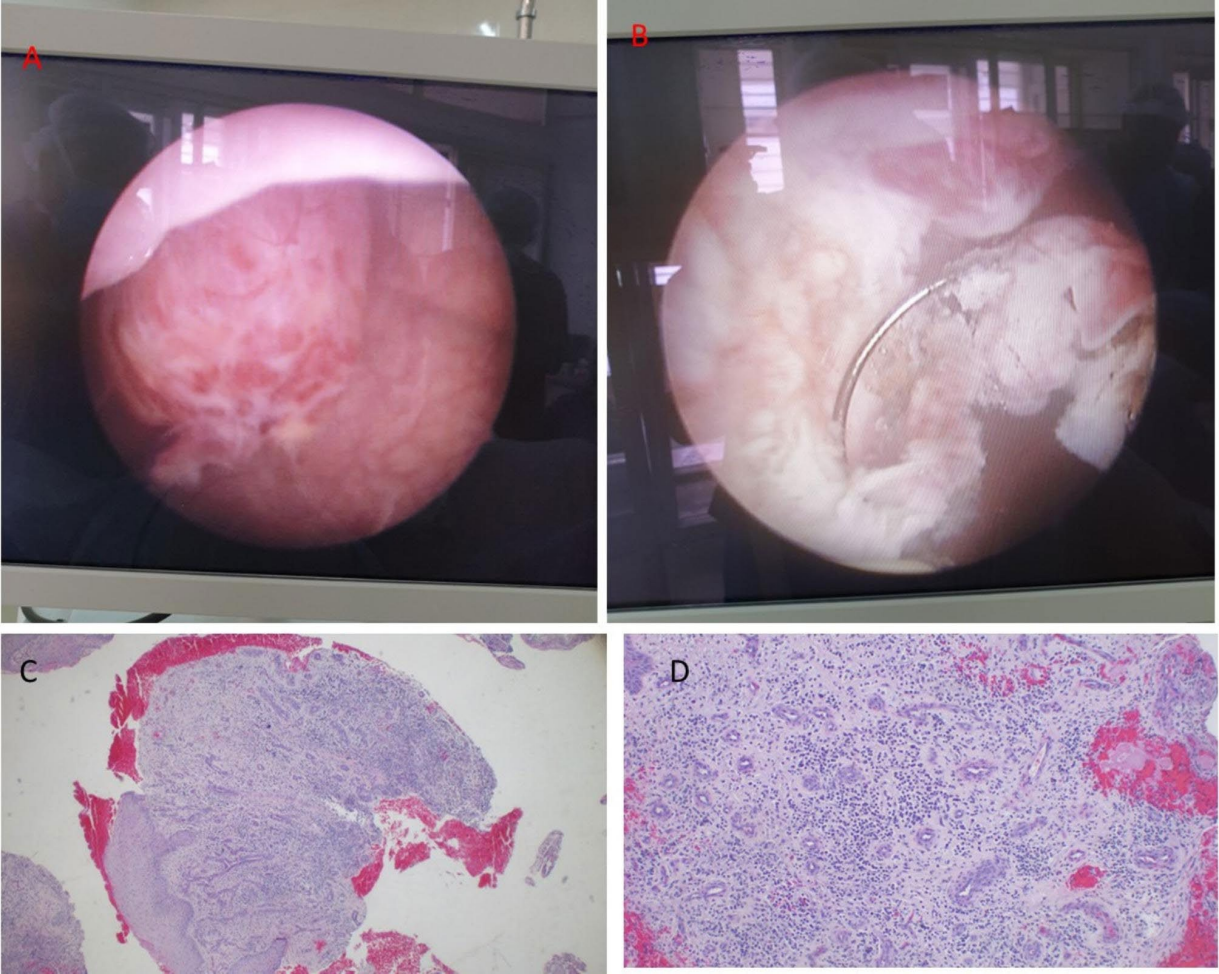
Clinical photograph showing a sinus discharging pus (arrow) with surrounding scar tissue

**Fig. 3:**
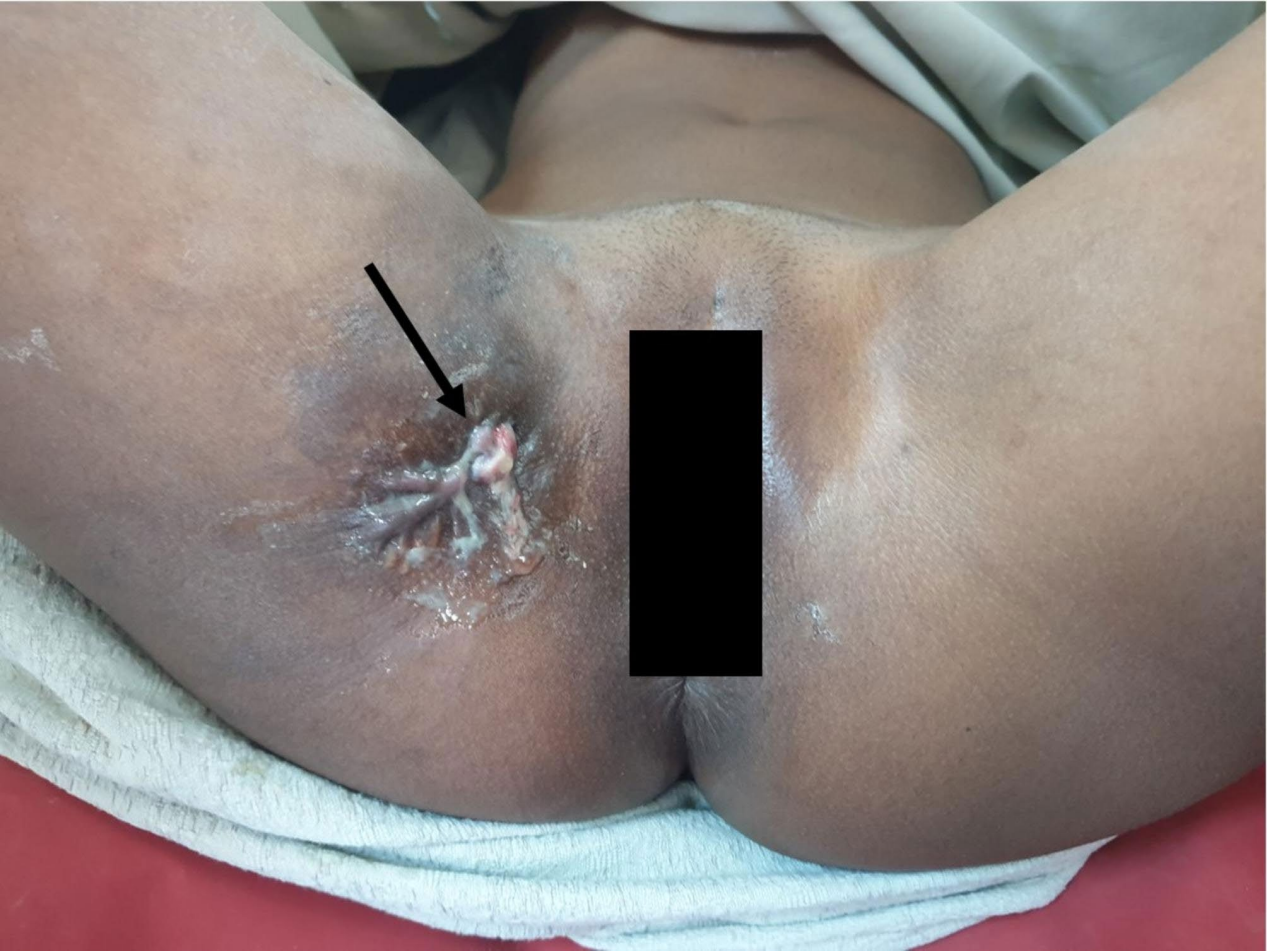
A: Granulomatous induration at 10 o’clock B: Resection using monopolar TUR set C: Pyogenic granuloma highlighting a highly vascularized proliferation of granulation tissue, ulceration and lobular arrangement (H&E stain, 40× original magnification) D: Presence of dense sub-acute inflammatory cell infiltrate comprised of neutrophils, lymphocytes and plasma cells (H&E stain, 100× original magnification)

Laboratory work up showed hemoglobin of 11.5 g/dL, serum creatinine of 43 mmol/l, BUN of 1.95 mmol/l and serum potassium and sodium within range. She was scheduled for cystoscopy, exploration and fistulectomy. On examination under anesthesia she was noted to have normal appearing external female genitalia, and a 1 by 1 cm sinus on her upper thigh in the inner aspect, with exophytic edge, discharging pus and surrounded by firm scar tissue (Fig. 2). Cystoscopy revealed a 2 by 2 cm mass at 10 o’clock approximately 5 cm away from the trigone that was easily bleeding to tough. The mass was resected off the wall using monopolar TUR set and the fistula opening was appreciated (Fig. 3), a 2-way 16Fr Foley catheter was left in situ and the tissue chips were sent for histopathology which revealed pyogenic granuloma (Fig. 3).

**Fig. 4:**
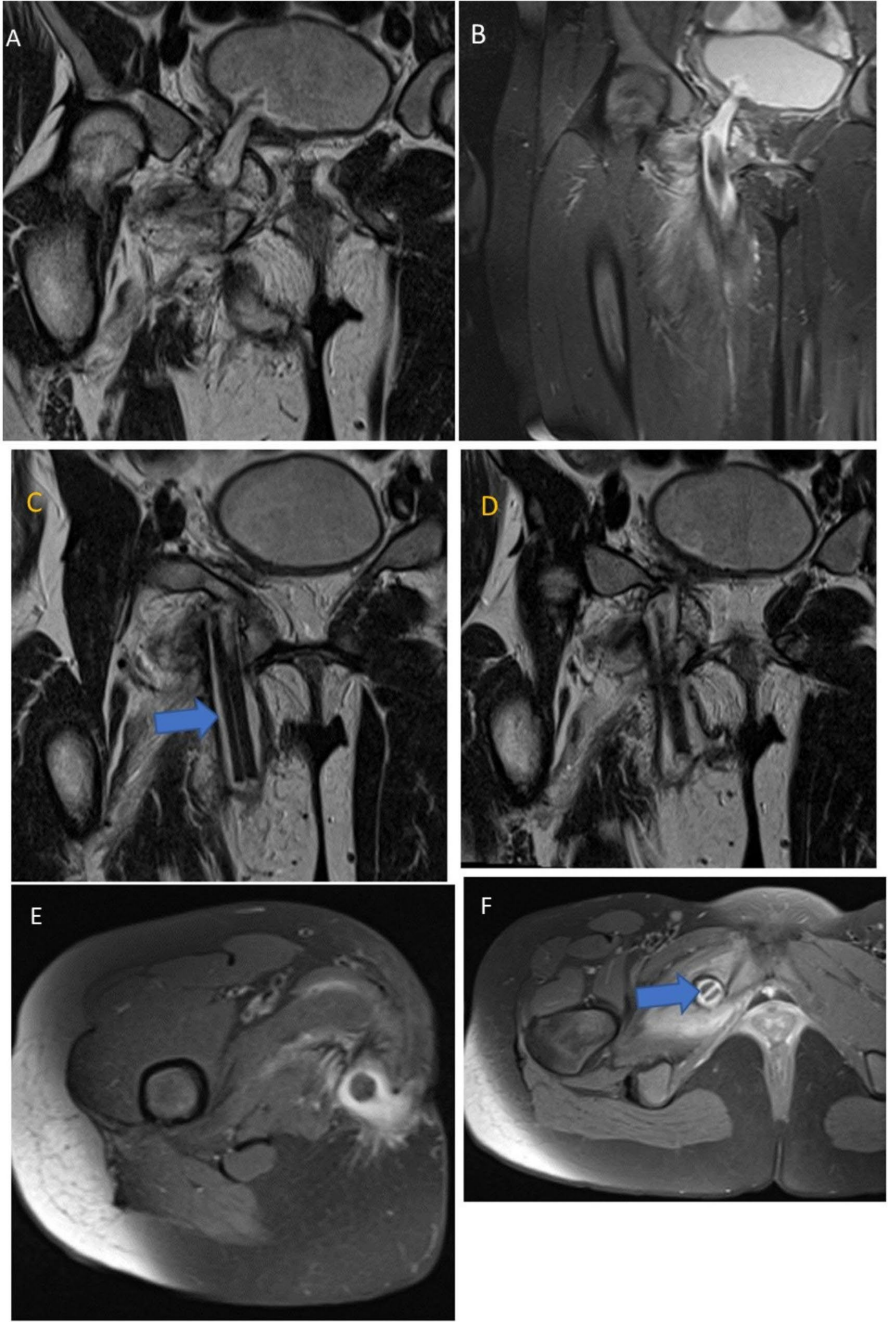
Clinical photographs showing a 10 cm wooden stick impacted within the fistula tract

On exploration of the sinus, incision was made around the fistula, externally, few centimeters from the center and extended upwards about 2 cm. A foreign body was felt with forceps that seemed to project in the direction of right lateral wall of the urinary bladder through muscle layers. On controlled division and layered dissection a round old piece of wood measuring about 7 cm long with an estimated diameter of 6 mm was extracted out with no calcifications around (Fig. 4). The fistula tract was curated and debrided, thoroughly lavaged, hemostasis achieved and wound was dressed up aseptically. Tissue sample from the fistula tract was taken for histology analysis which confirmed pyogenic granuloma and no features of metastasis.

**Fig. 5:**
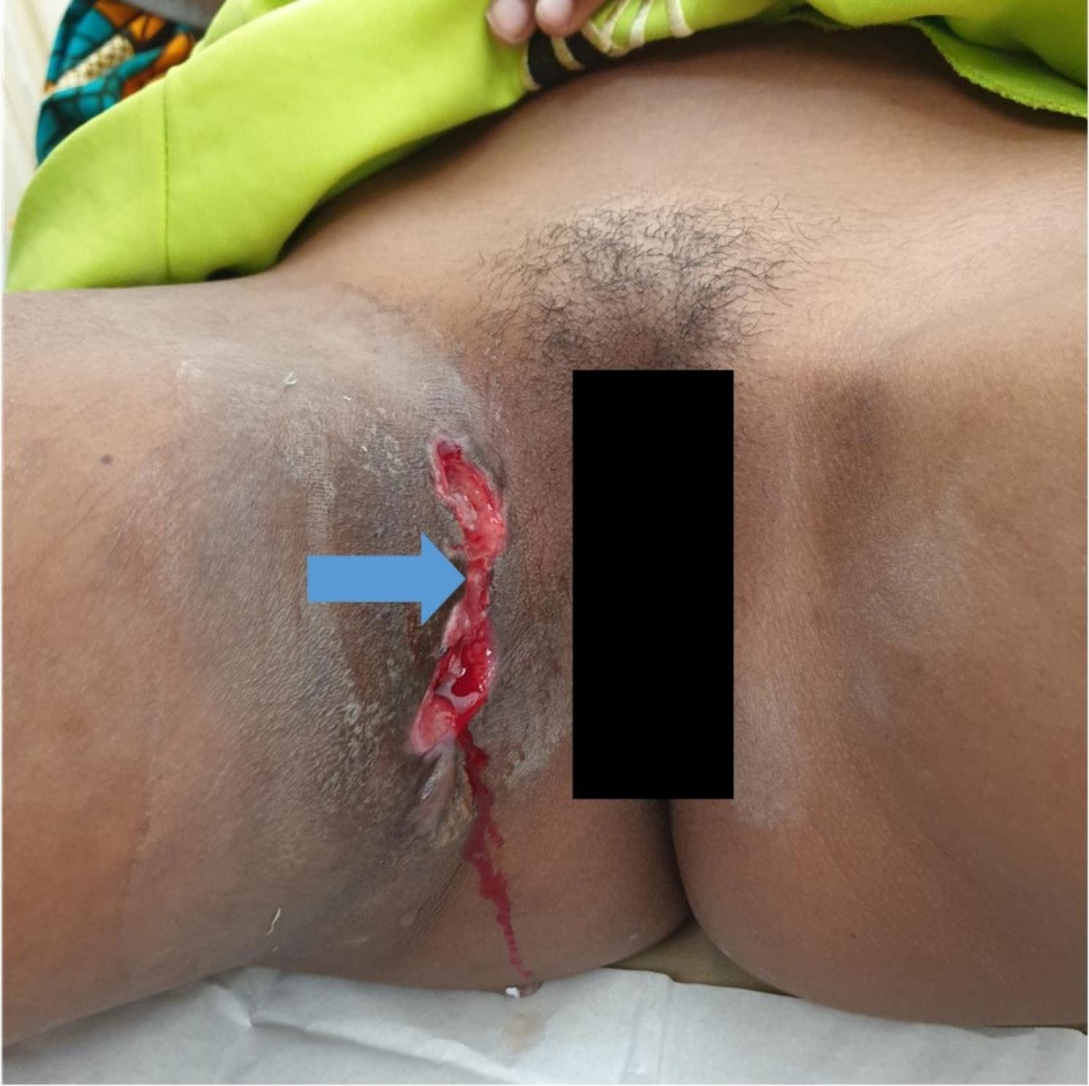
Clinical photograph showing healing sinus tract (Blue arrow)

Post operatively she was kept on Sitz-baths, a course of antibiotics and analgesics with no leakage of clear fluid/urine from the fistula tract or any adverse event noted. On day 21st post operatively the urethral catheter was removed and she continued to fair well (Fig. 5).

## Discussion

Urinary bladder trauma can be broadly classified as blunt or penetrating with blunt injury being the most common type. Penetrating injury to the bladder is relatively rare and accounts for 14–49%. Clinical presentation of bladder injury includes suprapubic pain, inability to pass urine, hematuria and peritoneal sign and symptoms in case of intraperitoneal injury [6]. The index case is unique as there was no reported sign and symptoms suggestive of bladder injury.

Port of entry can either be abdomen, perineum, rectum, buttocks or pelvis with upper inner thigh as the port of entry being a very rare presentation [1, 6]. Our case was diagnosed with penetrating bladder injury after a year and six months because of the unusual presentation of both symptoms and signs and the entry point. Complications of bladder injury includes persistent hematuria, infection (sepsis) and urine incontinence (fistula formation) [6, 7]. Pererira et al. also state in their literature that penetrating trauma to the lower abdomen, thighs or perineum is sensitive to bladder injuries [2]. Common cause of penetrating bladder injury is gunshot wounds (GSW) accounting for 80% versus 20% from stab wounds. One cannot predict the trajectory of a gunshot wound whereas stab wounds can be predicted with relatively little damage to surrounding tissues compared to GSW [8]. Other causes include pelvic fractures and isolated bladder injuries from iatrogenic injury, though are uncommon [8].

Diagnostic modalities for urinary bladder injuries can be X-ray cystography but CT-cystography is recommended if there is suspicion for other viscera injuries, provides a 3D image for evaluation, whereas on the contrary has exposure to more radiation [8]. Acute bladder injuries should be graded according to the Organ Injury Scale developed by the American Association of Surgery for Trauma (AAST) but this was not possible in the index case due to the chronicity of the injury and process of healing [8].

Thigh abscess associated with a fistula due to abdominal pathology has been reported in literature. VCF presenting as a thigh abscess can have various aetiologies such as extensive pelvic fracture, pelvic abscess, post pelvic radiation giant bladder calculus, IBD and post operative cases such as hip arthroplasty [9]. It is evident that VCF occur due to reaction from a foreign body as mentioned by Apul Goel et al. and Raghavendran et al. [10, 11]. Urinary fistula especially long standing can cause considerable physical and psychological stress, such as in the index case where she had to miss school due to recurrent thigh abscess. This is more likely where the cause cannot be ascertained and often misdiagnosed as in the index report [4].

Vesicocutaneous fistulas rarely communicate to the thigh but commonly exit the perineum, scrotum, buttocks or hypogastrium [12]. The time for the fistula to develop can be variable but usually precede an abscess [12]. We believe our patient did not leak urine because the fistula tract was blocked by the foreign body. For simple fistula a micturating cystourethrogram can be sufficient but in cases where the history is prolonged and fistula is complex a CT or an MRI are needed as evident in our case where the history was long and presentation was rather uncommon [13].

Management depends on the presenting symptoms, the complexity of the fistula and the underlying cause [14]. Urinary diversion to reduce the hydrostatic pressure of the bladder is vital therefore urethral catheter was kept for 21 days in our case. However, fistula excision surgery was not needed as the tract healed spontaneously after removal of the foreign object (causal agent) [3, 9]. VCF can also be managed surgically by fistulectomy with vacuum-assisted closure or larger and complex fistulas may require musculocutaneous flap surgery [15].

## Conclusion

Physician should always remember the possibility of having bladder injury and associated complications including fistula in a patient with history of penetrating trauma on the inner aspect of the upper thigh. A delayed presentation of the urinary tract fistula can occur hence a high index of suspicion is required for early diagnosis and prompt management. This case report highlights the uncommon cause and the aid of radiology imaging in yielding the diagnosis.

## Data Availability

Not applicable.

## Abbreviations

CT: Computed tomography
IBD: Inflammatory bowel disease
MRI: Magnetic resonance imaging
